# Supplementary material for: Real-World Treatment Patterns and Timeliness of Clinical Care Pathway for Non-Small Cell Lung Cancer Patients in Austria: The PRATER Retrospective Study
Source: Cancers (Basel). 2024 Jul 19;16(14):2586. doi: 10.3390/cancers16142586 (PMC11275022; doi:10.3390/cancers16142586)
Supplement: Supplementary file 1 [file cancers-16-02586-s001.zip › cancers-3077650-supplementary.pdf]

Supplementary Materials – Contents

Supplementary Methods 2
Supplementary Tables 3
Supplementary Figures 6

Supplementary Methods

Rationale for study-specific definition of COVID-19 period:

According to the World Health Organization (WHO), COVID-19 was declared a Public Health Emergency of International Concern (PHEIC) on 30-January-2020 and the outbreak was characterized a pandemic on 11-March-2020. As of 5-May-2023, this well-established and ongoing disease is no longer considered a PHEIC (https://www.who.int/europe/emergencies/situations/covid-19#:~:text=On%205%20May%202023%2C%20more,the%20definition%20of%20a%20PHEIC).

However, at the time of study design and protocol preparation (year 2022), the pandemic was ongoing. Therefore, a cut-off at 31-December-2021 was set in order to obtain two equal time periods, spanning 2 years each, for the purposes of comparing NSCLC data between the pre-pandemic (2018-2019) and pandemic (2020-2021) periods. This was particularly important since the number and stage distribution of new NSCLC cases was planned to be assessed as a measure of impact of the pandemic on NSCLC care in Austria. Notably, this segregation also allowed for collection of data preceding the incorporation of new TT/ICI therapies in the routine care of early-stage NSCLC, which would otherwise heavily confound any conclusions with regards to the impact of the pandemic on systemic treatment rates.

Supplementary Tables

Table S1. Methodology for biomarkers examined in ≥20.0% of patients (excluding PD-L1), among patients with early-stage NSCLC who underwent specific biomarker testing, overall and per NSCLC stage at initial diagnosis.

		Overall (N=145)	Stage I (N=45)	Stage II (N=40)	Stage III (N=60)
**ALK**		**82 (56.6%)**	**28 (62.2%)**	**23 (57.5%)**	**31 (51.7%)**
	IHC	*57 (69.5%)*	*21 (75.0%)*	*11 (47.8%)*	*25 (80.6%)*
	NGS	*19 (23.2%)*	*4 (14.3%)*	*10 (43.5%)*	*5 (16.1%)*
	IHC & NGS	*3 (3.7%)*	*1 (3.6%)*	*1 (4.3%)*	*1 (3.2%)*
	IHC & NGS & Other	*2 (2.4%)*	*2 (7.1%)*	.	.
	Other	*1 (1.2%)*	.	*1 (4.3%)*	.
**ROS1**		**69 (47.6%)**	**23 (51.1%)**	**19 (47.5%)**	**27 (45.0%)**
	IHC	*47 (68.1%)*	*15 (65.2%)*	*10 (52.6%)*	*22 (81.5%)*
	NGS	*16 (23.2%)*	*4 (17.4%)*	*8 (42.1%)*	*4 (14.8%)*
	Other	*3 (4.3%)*	*1 (4.3%)*	*1 (5.3%)*	*1 (3.7%)*
	IHC & NGS & Other	*2 (2.9%)*	*2 (8.7%)*	.	.
	IHC & NGS	*1 (1.4%)*	*1 (4.3%)*	.	.
**EGFR**		**55 (37.9%)**	**17 (37.8%)**	**21 (52.5%)**	**17 (28.3%)**
	NGS	*29 (52.7%)*	*9 (52.9%)*	*12 (57.1%)*	*8 (47.1%)*
	IHC	*19 (34.5%)*	*5 (29.4%)*	*6 (28.6%)*	*8 (47.1%)*
	Other	*7 (12.7%)*	*3 (17.6%)*	*3 (14.3%)*	*1 (5.9%)*
**BRAF**		**48 (33.1%)**	**15 (33.3%)**	**18 (45.0%)**	**15 (25.0%)**
	NGS	*28 (58.3%)*	*9 (60.0%)*	*13 (72.2%)*	*6 (40.0%)*
	IHC	*15 (31.3%)*	*4 (26.7%)*	*3 (16.7%)*	*8 (53.3%)*
	Other	*5 (10.4%)*	*2 (13.3%)*	*2 (11.1%)*	*1 (6.7%)*
**KRAS**		**45 (31.0%)**	**13 (28.9%)**	**17 (42.5%)**	**15 (25.0%)**
	NGS	*26 (57.8%)*	*7 (53.8%)*	*12 (70.6%)*	*7 (46.7%)*
	IHC	*15 (33.3%)*	*4 (30.8%)*	*4 (23.5%)*	*7 (46.7%)*
	IHC & NGS	*1 (2.2%)*	.	*1 (5.9%)*	.
	Other	*3 (6.7%)*	*2 (15.4%)*	.	*1 (6.7%)*
**HER2**		**34 (23.4%)**	**10 (22.2%)**	**14 (35.0%)**	**10 (16.7%)**
	NGS	*26 (76.5%)*	*7 (70.0%)*	*12 (85.7%)*	*7 (70.0%)*
	IHC	*5 (14.7%)*	*1 (10.0%)*	*2 (14.3%)*	*2 (20.0%)*
	Other	*3 (8.8%)*	*2 (20.0%)*	.	*1 (10.0%)*
**NRTK**		**31 (21.4%)**	**11 (24.4%)**	**10 (25.0%)**	**10 (16.7%)**
	NGS	*17 (54.8%)*	*4 (36.4%)*	*7 (70.0%)*	*6 (60.0%)*
	IHC	*12 (38.7%)*	*6 (54.5%)*	*2 (20.0%)*	*4 (40.0%)*
	Other	*2 (6.5%)*	*1 (9.1%)*	*1 (10.0%)*	.
**MET**		**21 (14.5%)**	**6 (13.3%)**	**11 (27.5%)**	**4 (6.7%)**
	NGS	*16 (76.2%)*	*4 (66.7%)*	*9 (81.8%)*	*3 (75.0%)*
	IHC	*2 (9.5%)*	.	*1 (9.1%)*	*1 (25.0%)*
	Other	*2 (9.5%)*	*1 (16.7%)*	*1 (9.1%)*	.
	NGS & Other	*1 (4.8%)*	*1 (16.7%)*	.	.
**RET**		**19 (13.1%)**	**5 (11.1%)**	**10 (25.0%)**	**4 (6.7%)**
	NGS	*16 (84.2%)*	*5 (100.0%)*	*8 (80.0%)*	*3 (75.0%)*
	IHC	*2 (10.5%)*	.	*1 (10.0%)*	*1 (25.0%)*
	Other	*1 (5.3%)*	.	*1 (10.0%)*	.

Abbreviations: ALK, Anaplastic Lymphoma Kinase; EGFR, Epidermal Growth Factor Receptor; HER2, Human Epidermal Growth Factor Receptor 2; IHC, Immunohistochemistry; KRAS, Kirsten Rat Sarcoma Virus; N, number of patients with available data (i.e., tested for specific biomarker); NGS, Next-Generation Sequencing; NRTK, Neurotrophic Receptor Tyrosine Kinase; NSCLC, Non-Small Cell Lung Cancer; PD-L1, Programmed Death Ligand 1.

Table S2. Reasons that led to the suspicion of the disease or NSCLC diagnosis.

n (%)	**Overall (N=319)**
Incidental medical finding **(only)**	153 (48.0%)
Symptoms **(only)**	102 (32.0%)
Lung cancer screening **(only)**	41 (12.9%)
Symptoms **&** Incidental medical finding	9 (2.8%)
Symptoms **&** Lung cancer screening	6 (1.9%)
During admission in the emergency room **(only)**	3 (0.9%)
Incidental medical finding **&** During admission in the emergency room	3 (0.9%)
Symptoms **&** During admission in the emergency room	2 (0.6%)

Abbreviations: N, number of patients with available data; n, number of patients with variable; NSCLC, Non-Small Cell Lung Cancer.

Table S3. Time (days) from the visit that led to NSCLC diagnosis until start of initial therapeutic strategy for early-stage NSCLC, per insurance type and gender.

**Private insurance**			
	**1st visit ↓ Histological diagnosis**	**Histological diagnosis ↓ Start of initial therapeutic strategy**	**1st visit ↓ Start of initial therapeutic strategy**
Yes, N	24	24	24
Median (IQR)	23.0 (9.5-44.5)	13.5 (0.0-30.0)	37.0 (25.5-67.0)
Mean (SD)	46.0 (76.7)	21.4 (29.2)	67.4 (79.7)
No, N	150	145	145
Median (IQR)	20.0 (7.0-39.0)	23.0 (10.0-37.0)	50.0 (31.0-67.0)
Mean (SD)	26.8 (27.2)	26.0 (25.1)	53.2 (31.1)

**Gender**				
	**1st visit ↓ 1st imaging**	**1st imaging ↓ Histological diagnosis**	**Histological diagnosis ↓ Start of initial therapeutic strategy**	**1st visit ↓ Start of initial therapeutic strategy**
Male, N	164	164	157	157
Median (IQR)	0.0 (-7.0-2.5)	27.0 (13.5-47.5)	26.0 (13.0-43.0)	58.0 (41.0-81.0)
Mean (SD)	12.6 (81.0)	43.8 (65.8)	29.9 (27.4)	88.0 (94.4)
Female, N	146	146	141	141
Median (IQR)	0.0 (-13.0-5.0)	28.0 (13.0-53.0)	21.0 (2.0-34.0)	53.0 (31.0-80.0)
Mean (SD)	26.6 (168.0)	44.7 (72.9)	22.9 (23.6)	93.0 (164.2)

Abbreviations: IQR, Interquartile Range; N, number of patients with available data; NSCLC, Non-Small Cell Lung Cancer; SD, Standard Deviation.

Supplementary Figures

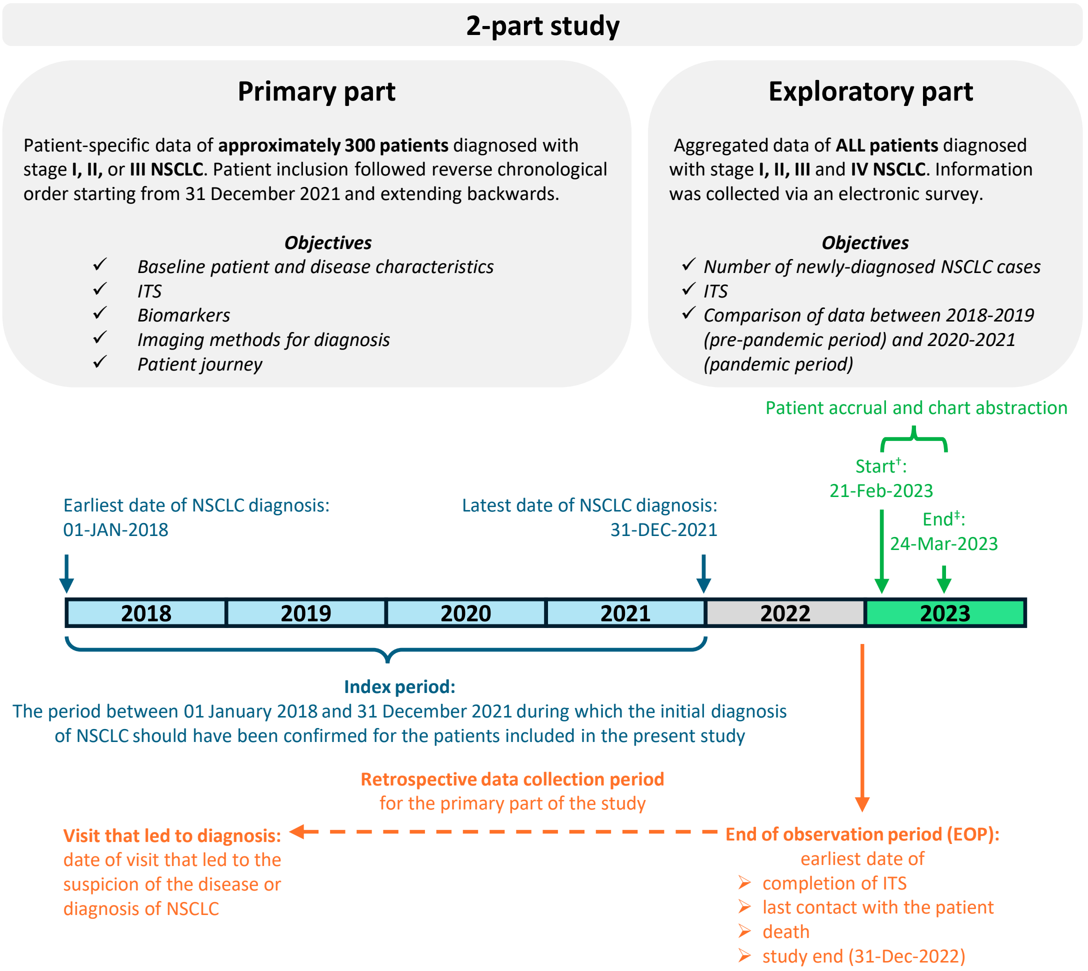

Figure S1. PRATER study design.

†First patient in.

‡Last patient in.

Abbreviations: EOP, End of Observation Period; ITS, Initial Therapeutic Strategy; NSCLC, Non-Small Cell Lung Cancer.

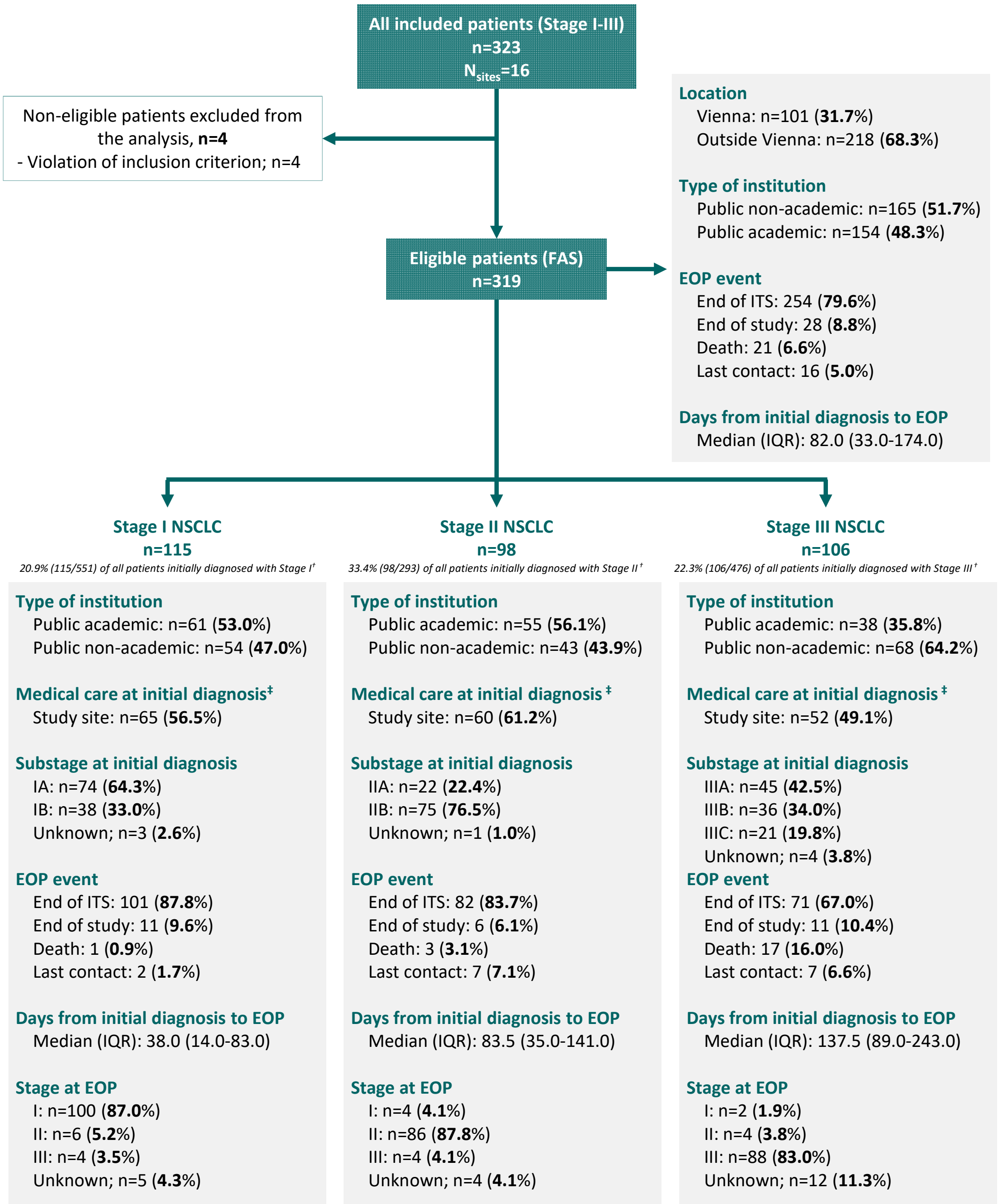

Figure S2. Disposition of patients included in analysis of individual patient-level data for patients initially diagnosed with stage I-III NSCLC.

Total of 16 study sites: 9 non-academic and 7 academic; 4 inside and 12 outside Vienna; 9 pneumologists, 3 oncologists, 3 thoracic surgeons and 1 pulmonologist.

†Corresponds to initial diagnoses during the site-specific index period (i.e., the period between the earliest and latest date of early-stage NSCLC diagnoses of the patients enrolled in the study by the specific site).

‡Healthcare setting of the medical visit that led to the suspicion of the disease or to NSCLC diagnosis.

Abbreviations: EOP, End of Observation Period; FAS, Full Analysis Set; IQR, Interquartile Range; ITS, Initial Therapeutic Strategy; N_{sites}, number of study sites with available data; n, number of patients with variable; NSCLC, Non-Small Cell Lung Cancer.

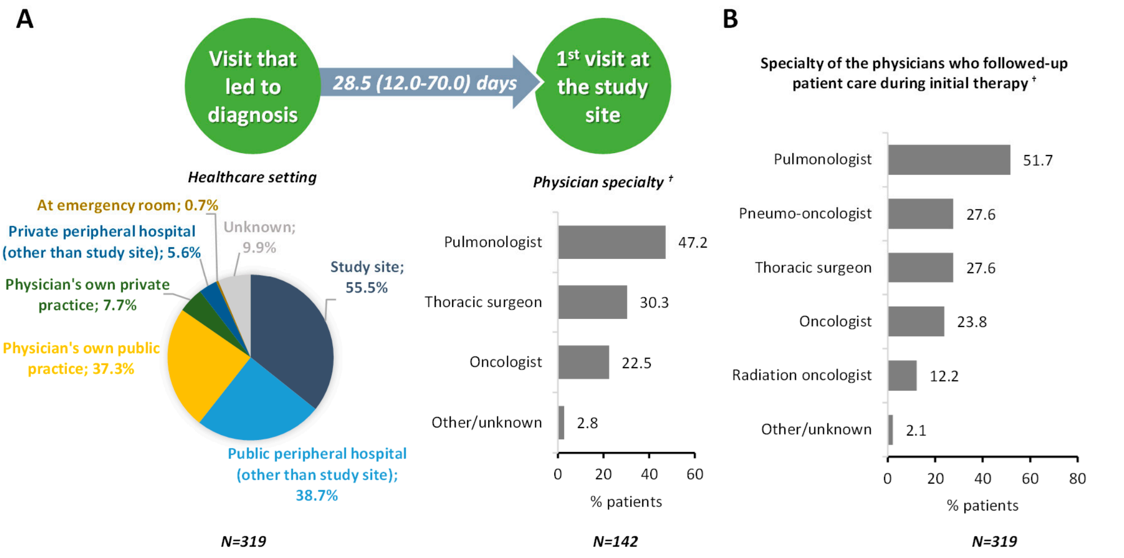

Figure S3. Healthcare setting and medical specialties involved in the patient journey, in the overall stage I-III population: (A) Healthcare setting of the visit that led to NSCLC diagnosis and specialty of the physician involved in the 1st visit at the study site, and (B) physician specialty who followed-up patient care during initial therapy.

Arrow indicates median (interquartile range) time for patients who were NOT under the care of the study site during the visit that led to NSCLC diagnosis.

†The sum of percentages exceeds 100 because in some cases more than one different physician specialties were involved.

Abbreviations: NSCLC, Non-Small Cell Lung Cancer.

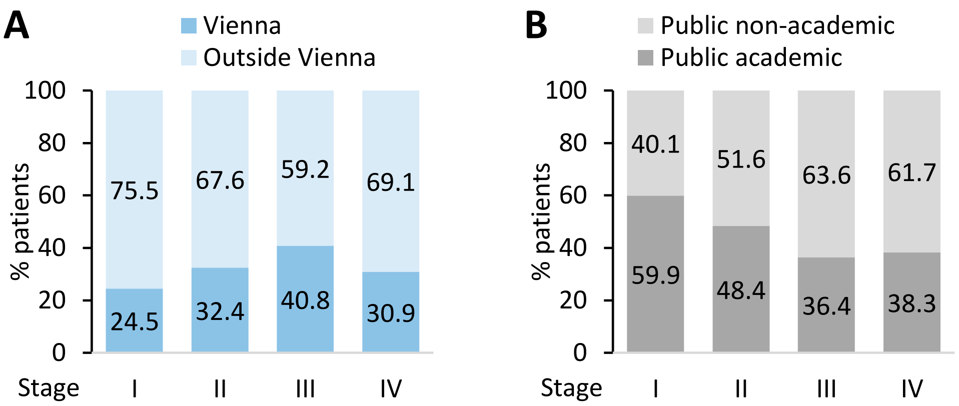

Figure S4. Results based on aggregate high-level data for patients initially diagnosed with stage I-IV NSCLC: patient disposition in terms of (A) study site location, and (B) institution type, per NSCLC stage at initial diagnosis.

Total of 14 study sites: 8 pneumologists, 3 oncologists, and 3 thoracic surgeons.
Abbreviations: NSCLC, Non-Small Cell Lung Cancer.
